# Effective intravitreal gene delivery to retinal pigment epithelium with hyaluronic acid nanospheres

**DOI:** 10.1016/j.omtn.2024.102222

**Published:** 2024-05-20

**Authors:** Ryan Crane, Mustafa S. Makia, Stephanie Zeibak, Lars Tebbe, Larissa Ikele, Christian Rutan Woods, Shannon M. Conley, Ghanashyam Acharya, Muna I. Naash, Muayyad R. Al-Ubaidi

**Affiliations:** 1 Department of Biomedical Engineering, University of Houston, Houston, TX 77204, USA; 2 Department of Cell Biology, University of Oklahoma Health Sciences Center, Oklahoma City, OK 73104, USA; 3 Department of Surgery, Baylor College of Medicine, Houston, TX 77030, USA; 4 College of Optometry, University of Houston, Houston, TX 77204, USA

**Keywords:** MT: Delivery Strategies, hyaluronic acid, retina, photoreceptor, retinal pigment epithelium, intravitreal injection, sulfotyrosine, inner limiting membrane

## Abstract

Inherited retinal degeneration (IRD) can cause a wide range of different forms of vision loss and blindness, and in spite of extensive advancements in gene therapy research, therapeutic approaches for targeting IRDs are still lacking. We have recently developed an approach for the intravitreal co-delivery of hyaluronic-acid nanospheres (HA-NSs) with sulfotyrosine (ST), effectively reaching the outer retina from the vitreal cavity. Here, our goal was to understand whether DNA-filled HA-NSs could generate gene expression in the outer retina. TxRed-labeled HA-NSs were compacted with plasmid DNA carrying a GFP reporter gene and intravitreally injected into the mouse retina. Follow-up at 4 weeks showed widespread gene expression in the outer retina and reduced, albeit present, expression at 8 weeks post-injection. Further analysis revealed this expression to be largely localized to the retinal pigment epithelium (RPE). These data show that intravitreal delivery of HA-NSs is a promising non-viral platform for the delivery of therapeutic genes and can generate pan-tissue, persistent gene expression in the RPE.

## Graphical abstract

**Figure:**
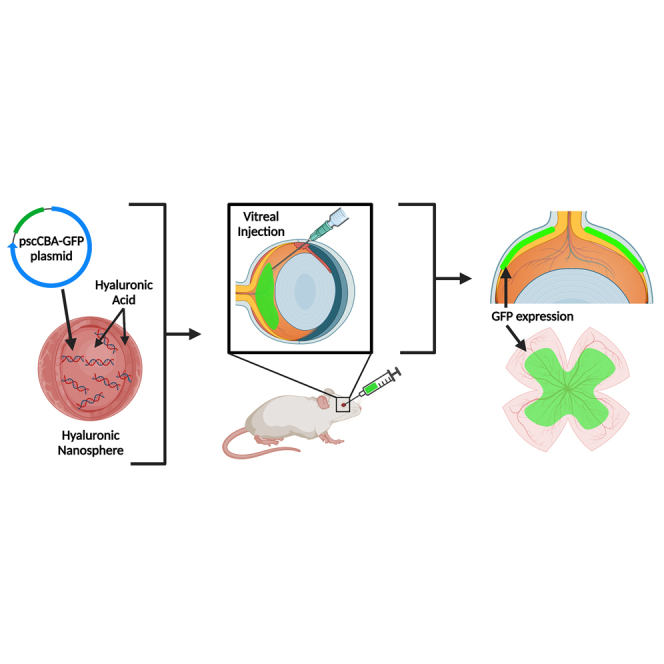

## Teaser

Al-Ubaidi and colleagues highlight the use of nanoparticles to successfully deliver plasmid DNA to the retina via the vitreous space in the eye, which then led to expression. This was achieved through the co-injection of a delivery agent, allowing for the passage of the nanoparticles into the retinal space.

## Introduction

Inherited retinal degeneration (IRD) results from mutations in one or more genes critical for retinal function, and the disease burden and severe loss of vision that accompany many of these mutations have made the development of effective genetic therapies a research priority. The most common IRD mutations are in genes expressed in the outer retina, specifically in the retinal pigment epithelium (RPE) and the photoreceptors.^1^ While many viral and non-viral genetic therapies have been evaluated in animal models, only one gene therapy has thus far been approved by the FDA for use in humans. Specifically, subretinal delivery of an adeno-associated virus (AAV) carrying RPE65 (Luxturna) has been approved to treat individuals suffering from Leber’s congenital amaurosis.^2^^,^^3^^,^^4^ Despite the initial success of the AAV-mediated RPE65 gene therapy, several challenges limit the widespread application of AAV. One of the biggest drawbacks is the limited carrying capacity of AAV, <5 kbp. This is particularly problematic since many retinal disease genes, such as those associated with Usher syndrome or Stargardt’s macular dystrophy, are too large to be delivered by AAV. While work is ongoing to develop new strategies to overcome the limitations of AAV,^5^ an alternative approach is the use of non-viral gene delivery mechanisms.

Non-viral gene delivery methods often involve complexing DNA with lipids, amino acids, or carbohydrates, and a wide variety of different non-viral gene therapies have been evaluated in the retina with varying degrees of success (reviewed in Zulliger et al.,^6^ Bisht et al.,^7^ and Conley and Naash^8^). One of the most well-explored options is a polyethylene glycol-linked polylysine (CK30PEG) nanoparticle, which generated persistent gene expression in the retina (and other tissues) and mediated phenotypic rescue in multiple IRD models.^9^^,^^10^^,^^11^^,^^12^ Other polymer-based gene therapy approaches have also showed promise for the delivery of large genes; for example, nanoparticles compacted with PEG and (1-aminoethyl)iminobis[N-(oleoylcysteinyl-1-amino-ethyl)propionamide have been used to improve phenotypes in the *Abca4*^−/−^ IRD model.^13^ However, similar to other gene therapy attempts, rescue with these nanoparticles was not complete and had limited efficiency outside of the injection site, largely due to its delivery via subretinal injection.^10^^,^^11^^,^^14^ Subretinal injections are the most effective way to deliver therapeutic agents directly to the outer retina. However, the procedure is invasive, results in retinal detachment, can trigger an inflammatory response,^15^ and often leads to restricted patterns of drug delivery limited to the area near the injection site. As a result, there has been interest in developing therapies that can be delivered via intravitreal injection. This method is widely used in animal models and in the clinic to target the inner retina^16^^,^^17^^,^^18^; however, the inner limiting membrane (ILM) has largely prevented effective delivery of nanosized materials to the outer retina following intravitreal delivery.^19^^,^^20^

Recently, we have begun to explore delivery approaches that permit materials to penetrate through the ILM to be delivered to the outer retina after intravitreal injection. One such method is co-delivery of hyaluronic acid (HA) nanospheres (HA-NSs) and sulfotyrosine (ST).^21^ Due to the high concentration of HA in the vitreous humor, the HA-NSs have minimal potential to induce adverse immune or inflammatory responses in the vitreous, and co-delivery of ST temporarily opens the ILM, likely by interacting with tyrosine-O-sulfated proteins, which are highly concentrated in the ILM and outer limiting membrane.^22^ After intravitreal co-injection of fluorescently labeled HA-NSs and ST, we observe robust delivery to the subretinal space.^21^ Here, we further explore the potential of these HA-NSs for the delivery of genetic material to the subretinal space. We find that for up to 8 weeks post-injection (the longest time examined), intravitreal delivery of HA-NSs carrying (chicken beta-actin) CBA-GFP generates widespread GFP expression in the RPE, suggesting that this approach may be an exciting new alternative for outer retina gene therapy.

## Results

### Characterization of HA-NSs

Previously, we evaluated the ability of fluorescently labeled HA-NSs with sizes of either 250 or 500 nm to reach the outer retina after intravitreal injection.^21^ Here, we assessed the ability of these HA-NSs in mediating gene expression after intravitreal delivery. We synthesized fluorescently tagged HA-NSs containing three different amounts of CBA-GFP plasmid DNA or without any DNA. The HA-NS diameter was measured by dynamic light scattering (DLS) and ranged from 52 nm for the empty HA-NSs (i.e., no DNA) to 226 nm for HA-NSs filled with the highest amount of DNA (102 ng DNA; Table 1; Figure S1A). The zeta potential (Table 1; Figure S1B) for the HA-NSs ranged from anionic for the empty HA-NSs (−44 mV) to nearly neutral for the HA-NSs carrying the largest amount of DNA (−0.63 mV). Measurements of the polydispersity index showed that all the HA-NSs maintained a value considerably below one regardless of the amount of packaged DNA (Table 1), suggesting that the HA-NSs maintained a good level of size uniformity. To visualize the NSs, they were filled with fluorescein isothiocyanate (FITC)-dextran and visualized with light microscopy (Figure S2A). To confirm that the DNA was contained within the NS and not leaking out, we pelleted the NS by centrifugation and measured the DNA in the supernatant. No DNA was detected in the supernatant (data not shown), suggesting it was well packaged. To further confirm the stability of the NSs and the confinement of the DNA within them, we separated HA-NSs with low, medium, and high amounts of DNA on 0.8% agarose gel. The results confirmed the integrity of the encapsulated DNA (Figure S2B). For assessing the stability of the HA-NSs, the HA-NS-high group was heated for 20 min at 55°C and then separated on an 0.8% agarose gel. The heated HA-NSs (Figure S2C) ran at a similar size to the unheated HA-NSs (Figure S2A) and did not show the smaller band characteristic of unpackaged naked DNA (Figure S2C, last lane), suggesting that the packaging was stable. To confirm that the HA-NSs could express the packaged DNA, the HA-NSs were transfected into HEK cells using lipofectamine 3000 (Figure S2D). The transfection rate for HA-NSs was lower than for naked DNA, likely due to the fact that the lipofectamine reagent is optimized for transfecting naked DNA rather than DNA encapsulated in nanoparticles. However, we did observe GFP expression in HA-NS-transfected cells, confirming that the encapsulated DNA could be expressed.

**Table 1:** HA-NSs were tagged with TxRed and then compacted with CBA-GFP plasmid as indicated in the table

	DNA concentration (ng/μL)	DNA/injection (ng)	Size (nm)	Zeta potential (mV)	PDI
HA-NS-empty	0	0	52.337 ± 0.492	−44.167 ± 2.581	0.181 ± 0.013
HA-NS-low	45.3	56.6	61.667 ± 0.701	−32.267 ± 2.318	0.217 ± 0.004
HA-NS-medium	65.4	81.7	132.200 ± 2.2070	−20.067 ± 0.651	0.163 ± 0.020
HA-NS-high	82.0	102.4	226.533 ± 11.719	−0.633 ± 0.643	0.294 ± 0.020

The amount delivered in each injection is in the second column. The HA-NS diameter was determined by performing dynamic light scattering measurements. Shown are mean values ± standard deviation. PDI, polydispersity index.

### GFP is expressed in the retina following intravitreal injection of HA-NSs

To evaluate the ability of HA-NSs to generate gene expression in the retina and the effects of co-delivery of ST, mice were intravitreally injected with 1.5 μL of either HA-NSs or HA-NSs plus ST. The amount of delivered DNA ranged from ∼50 (HA-NS-low) to ∼100 ng (HA-NS-high; Table 1), and we used ST at a concentration of 3.2 μg/μL, since this concentration previously improved retinal uptake of HA-NSs without inducing toxicity.^21^ Control mice were either saline injected or injected with uncompacted plasmid DNA (6.45 μg in 1.5 μL saline), an amount that we have previously shown to generate gene expression in the RPE after subretinal injection.^23^^,^^24^ At 4 weeks post-intravitreal injection (PI-4 weeks), a time point at which we previously observed the persistent presence of HA-NSs in the subretinal space,^21^ we carried out fundus imaging but observed no GFP signal above background in Figure S3.

**Figure 1:**
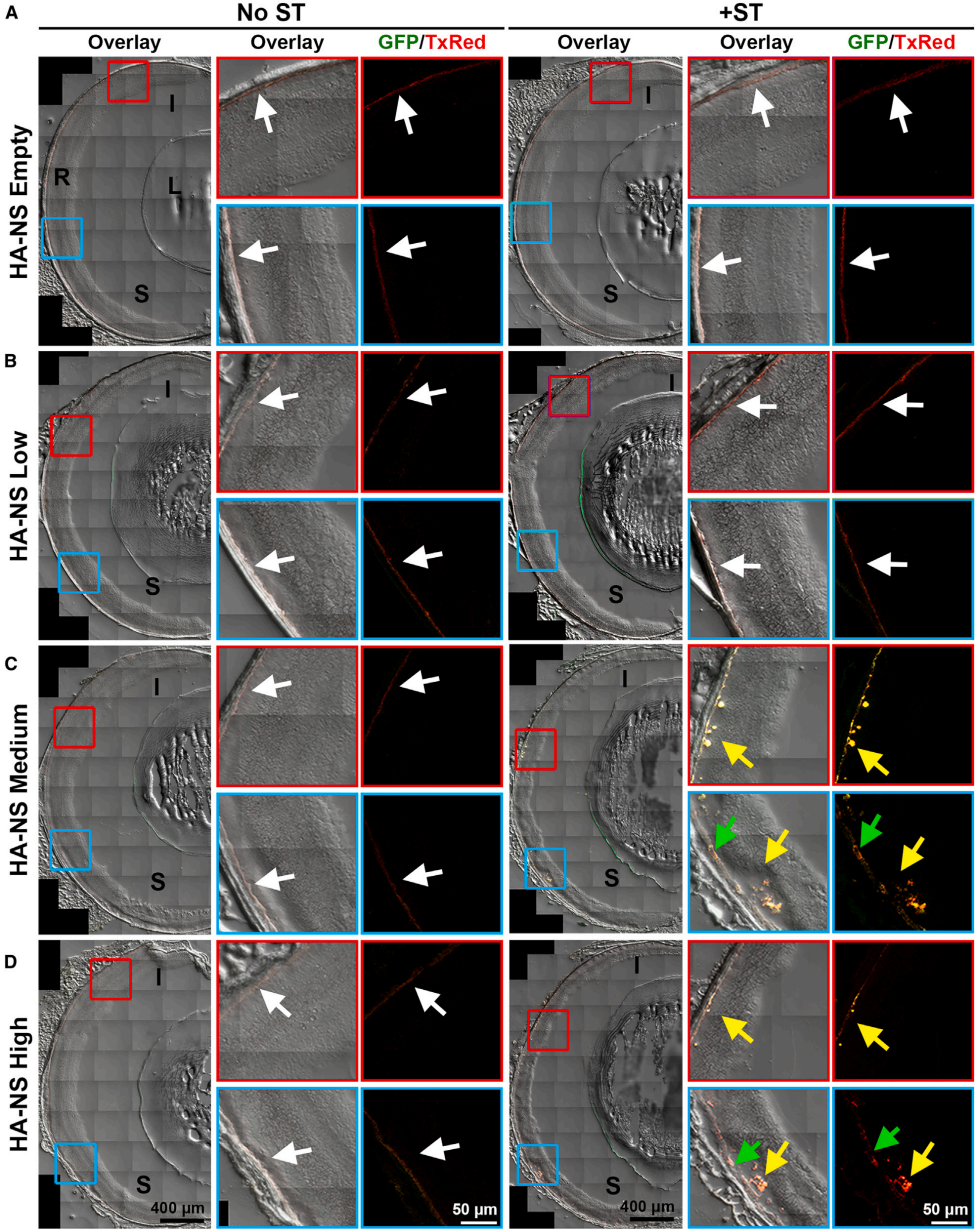
HA-NSs carrying CBA-GFP lead to gene expression at PI-4 weeks Eyes underwent intravitreal injection with HA-NS-empty (A), HA-NS-low (B), HA-NS-medium (C), or HA-NS-high (D), either with or without ST. At PI-4 weeks, eyes were harvested, cryosectioned, and imaged for native fluorescence. The left image of each image set shows the full retinal cross-section with bright-field, GFP, and TxRed signals (overlay). Red and blue boxed areas are shown larger in the middle (overlay) and right (GFP in green, TxRed in red) images. White arrows highlight TxRed expression and green arrows highlight GFP expression, while yellow arrows highlight the overlay of TxRed/GFP signals. Both eyes from two animals were collected and imaged per group. Scale bars: 400 μm for the overview images and 50 μm for insets. I, inferior; S, superior; R, retina; L, lens.

Since fluorescent fundus imaging has low sensitivity and resolution compared to post-mortem imaging by microscopy, eyes were cryosectioned and native fluorescence was imaged to evaluate the presence and distribution of both the HA-NSs (labeled with TxRed) and gene expression from the enclosed plasmid (GFP). At PI-4 weeks, we observed that the smaller-diameter HA-NSs (HA-NS-empty and HA-NS-low) were present within the subretinal space either with or without co-delivery of ST (white arrows highlight the red signal from HA-NSs in Figures 1A and 1B). However, we did not observe an appreciable GFP signal with either the HA-NS-empty or HA-NS-low. In the HA-NS-medium and -high groups that were not co-injected with ST, we observed a small amount of red signal in the subretinal space but little or no GFP (white arrows, Figures 1C and 1D). In contrast, in animals co-injected with ST and either HA-NS-medium or HA-NS-high, we observed a robust GFP signal and red HA-NS signal in the subretinal space (yellow arrows highlight orange/yellow colocalization of red and green signals, Figures 1C and 1D). This expression was largely localized to the RPE (examples highlighted with green arrows). We did not observe any red signal or GFP expression in the retina after intravitreal injection of either saline (vehicle) or naked (uncompacted) plasmid DNA with or without ST (Figure S4).

**Figure 2:**
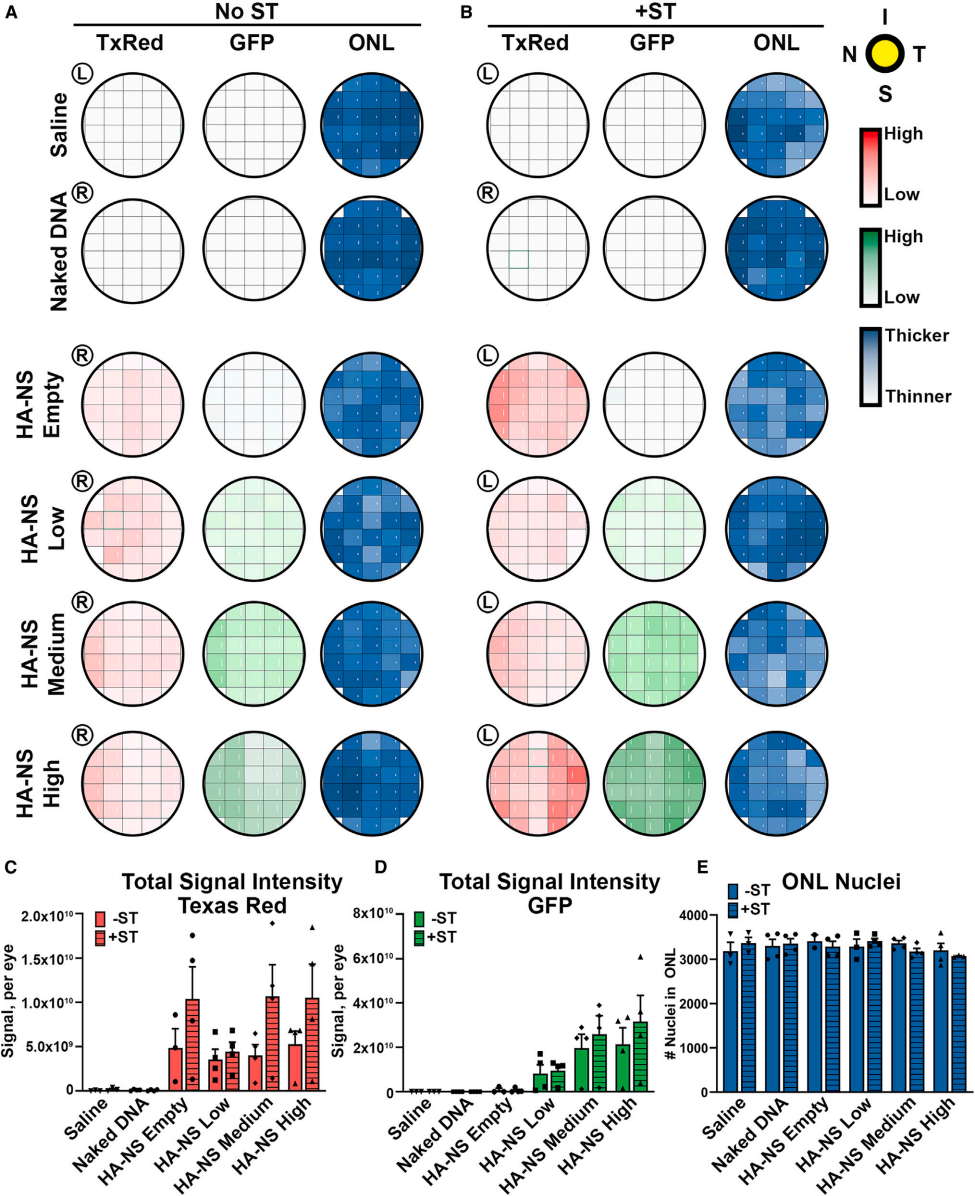
Co-delivery of ST with HA-NSs leads to gene expression without toxicity (A and B) Adult mouse eyes were injected with 1.5 μL of HA-NS-empty, HA-NS-low, HA-NS-medium, or HA-NS-high either without (A) or with (B) ST. Tissues were collected at PI-4 weeks and sectioned as in Figure S5. HA-NS TxRed fluorescence intensity and GFP fluorescence intensity were measured in adjacent regions throughout a retinal section and in multiple sections throughout the eye. Each row corresponds to a section, and each box corresponds to a region in that section (refer to Figure S5). Shown are maps of one eye per group, and additional eyes colored using the same criteria are shown in Figure S6. The intensity of color corresponds with increasing fluorescence intensity. Blue maps reflect the number of outer nuclear layer (ONL) nuclei in each region. (C–E) Total TxRed signals, GFP signals, and nuclei counts per eye were obtained by summing all the measured fields for each eye. Each symbol corresponds to one eye (*n* = 3–4/group). Both eyes from two animals were used for the analysis per group. Differences were assessed by two-way ANOVA; error bars are standard deviation; no significant differences were found between ST and no ST. R, right eye; L, left eye; I, inferior; S, superior; N, nasal; T, temporal.

To more accurately assess the distribution of HA-NSs and associated gene expression across the eye, we imaged sections throughout the eye along the nasal-temporal plane (example illustrated in Figure S5A). We then evaluated the total either red or green fluorescence signal from up to six fields of view (designated “regions,” Figures S5B–S5D) that made up each section. The fluorescence intensity values from each region were plotted as a heatmap in order to visualize distribution (Figures 2A and 2B, with additional eyes color coded using the same scale shown in Figure S6). More intense color in the heatmaps corresponds to a greater degree of fluorescence (red or green). As expected, eyes injected with HA-NS-empty did not show any appreciable GFP expression (Figures 2A and 2B), and overall, the amount of GFP expression increased with increasing amounts of DNA in the HA-NSs (Figures 2A and 2B). The TxRed and GFP signals were similarly distributed in eyes injected with HA-NSs carrying DNA, with the greatest levels of signal usually present in the more peripheral regions. However, critically, expression was widely distributed throughout the retina.

To better compare the overall levels of TxRed and GFP expression across groups, the signal intensity measurements from each region/section were summed to give a single value representing the whole eye, and then values from multiple eyes were plotted (Figures 2C and 2D). In most groups, there was a trend toward higher TxRed and GFP signals in eyes injected with ST, though the differences were not statistically significant.

To assess whether the HA-NSs were associated with retinal degeneration, photoreceptor nuclei (outer nuclear layer [ONL]) were counted across the eye to create similar maps (right columns in Figures 2A and 2B), with more intense blue corresponding to more ONL nuclei. We did not observe any patterns of retinal thinning that correlated with a TxRed or GFP signal, and when we plotted the total ONL nuclei counted/eye (Figure 2E), we did not observe any significant differences between groups, suggesting the HA-NSs and ST were well tolerated. This finding was supported by our observation that HA-NSs ± ST did not exert any toxic effects on cone or rod function as measured by electroretinogram (ERG; Figure S7).

### HA-NSs and associated GFP expression are detected in the outer retina at PI-8 weeks

**Figure 3:**
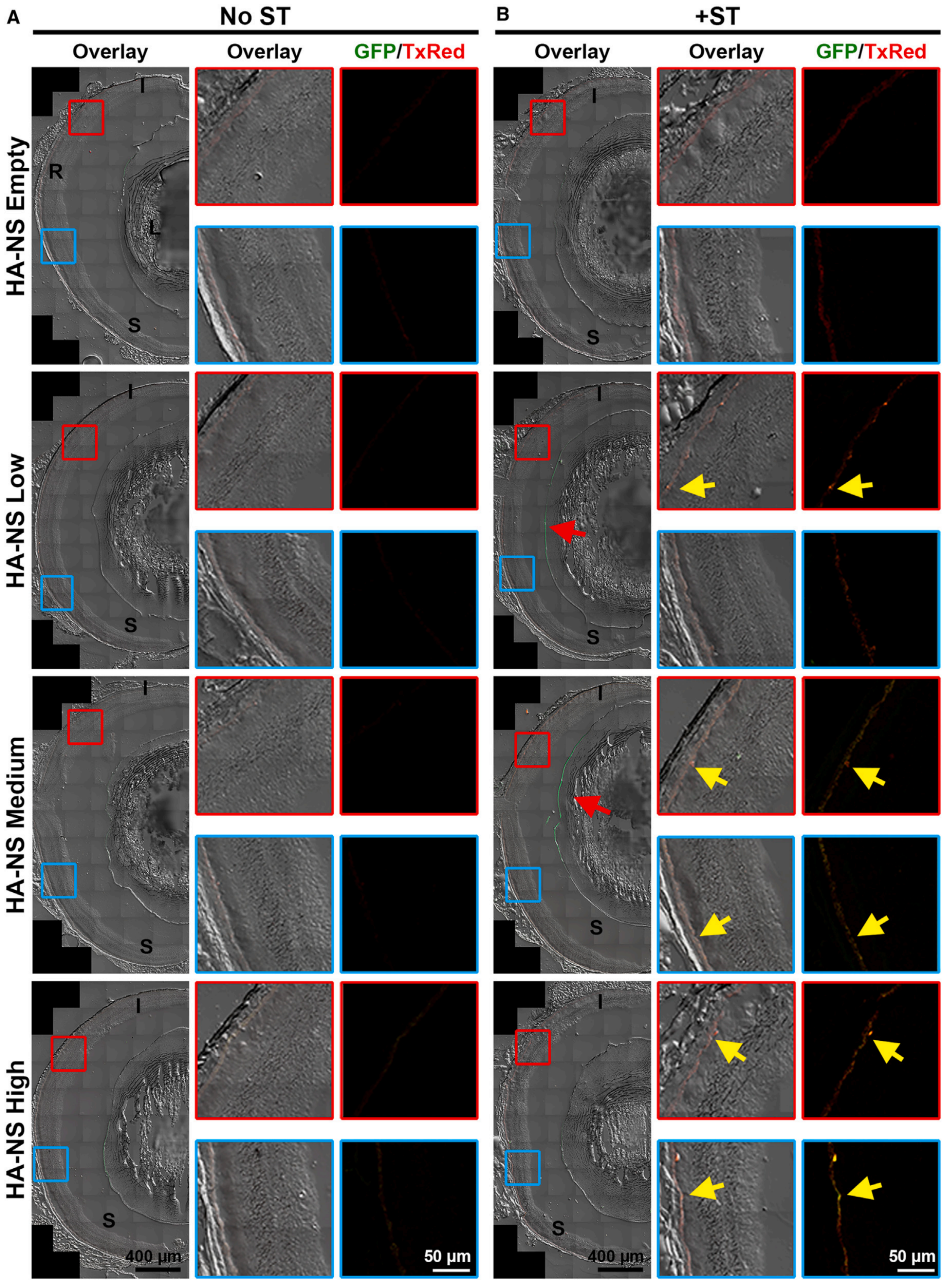
HA-NSs carrying CBA-GFP lead to gene expression at PI-8 weeks Eyes underwent intravitreal injection with HA-NS-empty, HA-NS-low, HA-NS-medium, or HA-NS-high either without (A) or with ST (B). At PI-8 weeks, eyes were harvested, cryosectioned, and imaged for native fluorescence. The left image of each image set shows the full retinal cross-section with bright-field, GFP, and TxRed signals (overlay). Red and blue boxed areas are shown larger in the middle (overlay) and right (GFP in green, TxRed in red) images. Red arrows highlight fluorescence along the ILM. One eye from one animal was collected and imaged per group. Yellow arrows highlight the overlay of TxRed/GFP signals. Scale bars: 400 μm for overview images and 50 μm for insets. I, inferior; S, superior; R, retina; L, lens.

**Figure 4:**
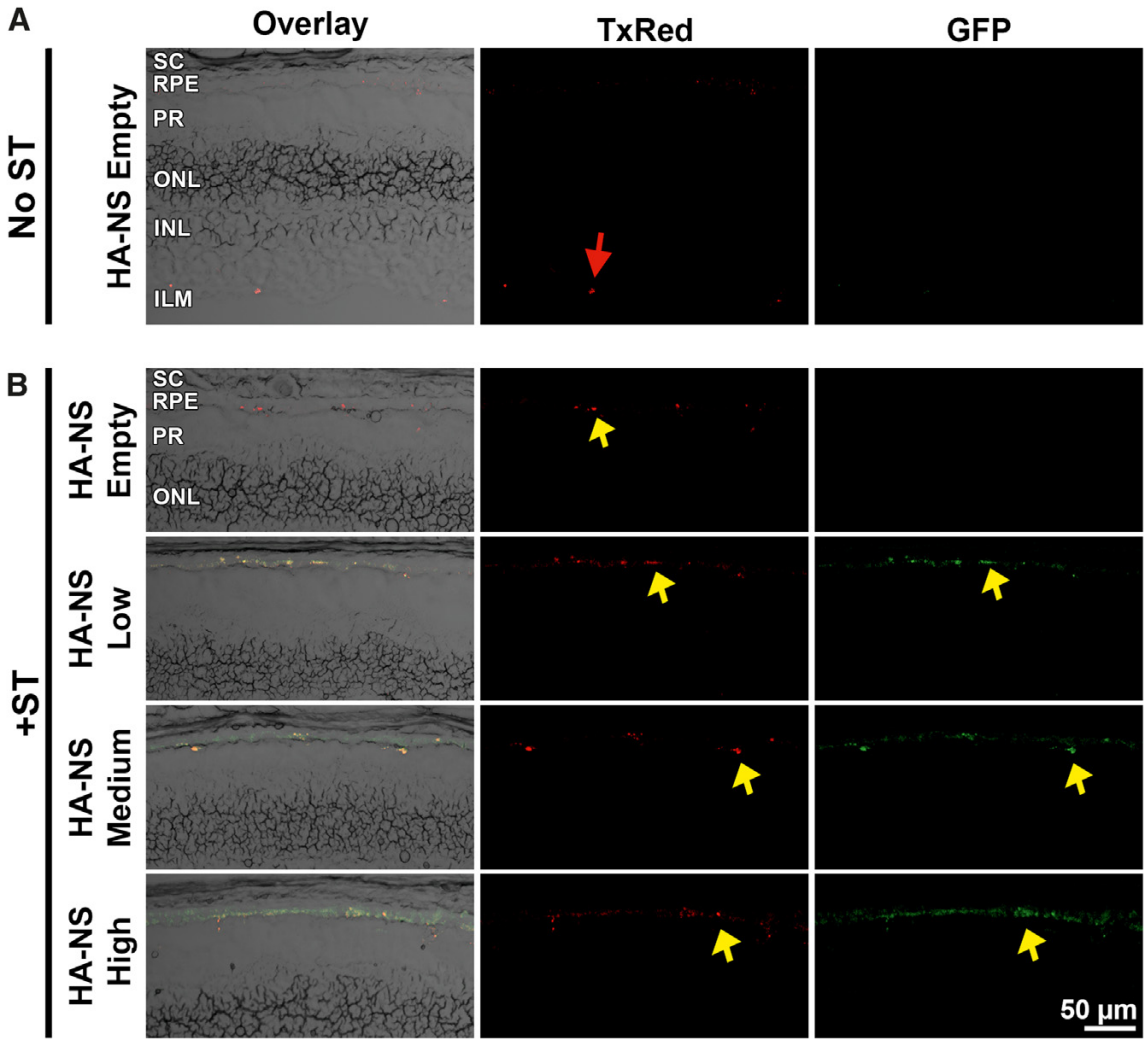
HA-NSs carrying CBA-GFP lead to gene expression in the outer retina at PI-8 weeks Eyes underwent intravitreal injection with HA-NS-empty, HA-NS-low, HA-NS-medium, or HA-NS-high either without (A) or with ST (B). Shown here are closeups of native fluorescence (and bright field) in eyes harvested at PI-8 weeks. The left image of each row shows the overlay with bright-field, GFP, and TxRed signals. Individual fluorescence channels are shown in the middle (TxRed) and right (GFP). Red arrow highlights HA-NSs along the ILM. One eye from one animal was collected and imaged per group. Yellow arrows highlight TxRed and GFP in the outer retina. Scale bars: 50 μm. SC, sclera; RPE, retinal pigment epithelium; PR, photoreceptor outer and inner segments; ONL, outer nuclear layer; INL, inner nuclear layer; ILM, inner limiting membrane.

**Figure 5:**
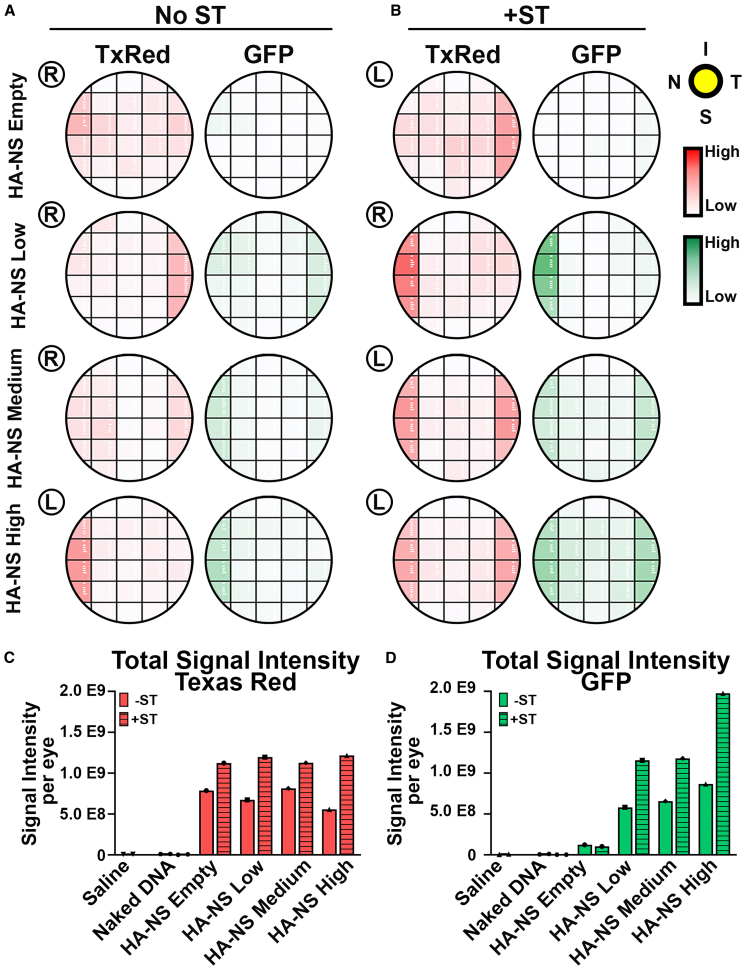
Co-delivery of ST with HA-NSs leads to widespread gene expression at PI-8 weeks (A and B) Adult mouse eyes were injected with HA-NS-empty, HA-NS-low, HA-NS-medium, or HA-NS-high either without (A) or with (B) ST. Tissues were collected at PI-8 weeks and sectioned as in Figure S5. HA-NS TxRed fluorescence intensity and GFP fluorescence intensity were measured in adjacent regions throughout a retinal section and in multiple sections throughout the eye. Each row corresponds to a section, and each box corresponds to a region in that section (refer to Figure S5). The intensity of color corresponds with increasing fluorescence intensity. Blue maps reflect the number of ONL nuclei in each region. (C and D) Total fluorescence intensity values per eye were obtained by summing all the measured fields for each eye. One eye from one animal was used for the analysis per group. R, right eye; L, left eye; I, inferior; S, superior; N, nasal; T, temporal.

After observing GFP expression in HA-NS-injected eyes at PI-4 weeks, we asked whether HA-NSs and associated GFP expression persisted for longer time points. A subset of animals was evaluated at PI-8 weeks. Fundus imaging was performed, however, as at PI-4 weeks, there was little or no observable GFP expression in the images (Figure S8), so we undertook fluorescent imaging of cryosectioned retinas. Little to no obvious GFP expression or HA-NSs were detected in the retinas of eyes injected without ST (Figure 3A). However, very rarely, we observed some HA-NSs remaining outside the retina adjacent to the ILM, as seen in Figure 4A (red arrow), which shows additional images of PI-8 week retinas with the red and green channels separated. In eyes co-injected with HA-NSs carrying DNA and ST, we observed persistent HA-NSs and GFP in the subretinal space (yellow arrows, Figures 3B and 4B). This GFP expression was most pronounced in HA-NS-high but was also observed in HA-NS-medium and HA-NS-low (heatmaps in Figures 5A–5D). The highest levels of GFP expression were seen in eyes injected with HA-NS-high, consistent with those particles containing the most DNA. At PI-8 weeks, GFP expression was still widely distributed throughout retinas injected with HA-NS-high and HA-NS-medium, while GFP expression was more localized in HA-NS-low (Figure 3B, yellow arrow). However, while GFP and TxRed were still detected at PI-8 weeks, the levels were approximately 10-fold less than what was seen at PI-4 weeks (Figures 5C, 5D, and S9, which shows total eye fluorescence for PI-4 weeks and PI-8 weeks plotted together).

**Figure 6:**
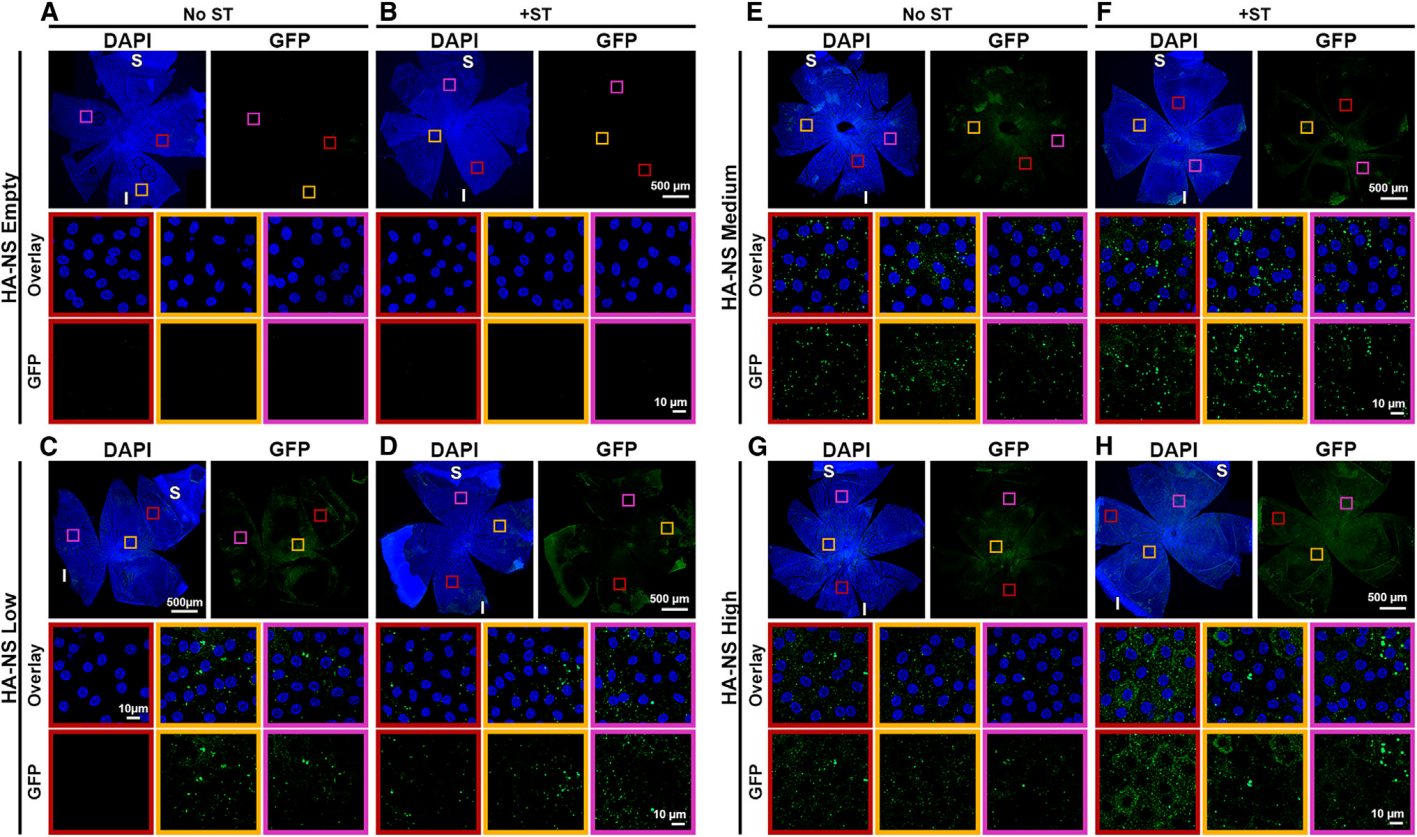
HA-NSs lead to widespread gene expression in the RPE Adult mouse eyes were injected with HA-NS-empty, HA-NS-low, HA-NS-medium, or HA-NS-high either without (A, C, E, and G) or with (B, D, F, and H) ST, and tissues were harvested at PI-8 weeks. Shown are RPE flat mounts (top row in each image) stained with GFP antibodies (green) and counterstained with DAPI (blue). Middle and bottom rows in each set of images show enlarged views of boxed regions in the top rows. One eye from one animal was collected and imaged per group. Scale bars: 500 μm for overview images and 10 μm for insets. S, superior; I, inferior.

While we saw GFP and HA-NS TxRed fluorescence in the subretinal space at PI-8 weeks, it was difficult to determine what cell type was expressing the GFP. Therefore, we performed retinal and RPE flat mounts and evaluated GFP expression by immunolabeling with an anti-GFP antibody. We did not observe any GFP signal in the neural retina (Figure S10). In contrast, we observed GFP expression in the RPE at PI-8 weeks in eyes injected with HA-NSs both with and without ST (Figure 6). The highest GFP signal in the RPE was in the HA-NS-high + ST group (Figure 6H), but a robust GFP signal was also detected in the HA-NS-medium groups (both with and without ST; Figures 6E and 6F). As expected, a GFP signal was not detected in HA-NS-empty (Figures 6A and 6B). No HA-NS red fluorescence was detected in any of the flat mounts, likely because the fixation and processing steps quenched the native fluorescence.

Mean signal intensities from regions of interest from RPE flat mounts of injected eyes indicate no differences in delivery efficacy between eyes injection with and without ST. However, given the high variability in signal intensities between samples, only eyes injected with NSs and ST passed statistical significance testing when compared to their corresponding negative controls.

## Discussion

Here, we provide additional evidence that the HA-NS delivery system is an effective tool for the delivery of material to the outer retina via intravitreal injection. Importantly, we also demonstrate that HA-NSs carrying plasmid DNA led to persistent (up to 8 weeks) gene expression in the RPE, while delivery of naked plasmid DNA led to no appreciable expression. Critically, the gene expression we observe after intravitreal injection is widely distributed throughout the retina and does not lead to damaging retinal detachment or retinal degeneration. As a result, this delivery approach may represent an exciting option in the retinal gene and drug delivery toolbox.

In the current study, we used a concentration of ST (3.2 μg/μL) that was the lowest dose we tested previously.^21^ Here, we found that this level of ST did not lead to any retinal degeneration, consistent with our prior study, in which we found that delivering lower doses (3.2 and 32 μg/μL) of ST and HA-NSs did not lead to retinal degeneration, impaired retinal function, or retinal inflammation.^21^ In that prior study, we did observe some toxicity with a dose that was 10-fold higher (320 μg/μL), thus generating a useful range of potential doses. Here, we observed that the co-delivery of ST did not significantly improve gene expression levels after delivery of HA-NSs. This may be due to insufficient ST and suggests that additional, future studies are needed to understand what potential role ST could play in the retinal uptake of HA-NSs.

One of our observations is that gene expression is limited to the RPE. One possibility for this is related to the timing of DNA release from the HA-NSs. If the DNA is released in the subretinal space, then the ability to transfect other retinal cells may be low. However, given that the GFP and HA-NS red fluorescence often co-localize in the RPE, a second option may be that the HA-NSs are taken up into RPE cells prior to releasing their DNA and that photoreceptors and other retinal neurons are not able to readily take up HA-NSs. RPE cells are highly phagocytic, and we have previously demonstrated that they robustly take up both naked plasmid DNA and CK30PEG10K nanoparticles from the subretinal space.^14^^,^^23^^,^^24^ While our current data suggest that these HA-NSs would be suitable for diseases associated with the RPE, such as Leber congenital amaaurosis (LCA), further exploration is needed to achieve photoreceptor transfection with this method. One key future step is evaluating and potentially altering the timing of DNA release from the HA-NS. However, even if HA-NSs are optimized to release their DNA cargo while in the photoreceptor/ONL rather than the subretinal space, that may be insufficient to generate robust photoreceptor transfection. We have observed that photoreceptors are not readily transfected by naked plasmid DNA, but they do take up CK30PEG10K nanoparticles.^9^^,^^10^^,^^11^ Therefore, one option for achieving photoreceptor transfection after intravitreal delivery would be to package CK30PEG10K nanoparticles into the larger HA-NSs and co-deliver them with ST.

Another area for further optimization is highlighted by our observation that GFP expression and HA-NS TxRed decreased from PI-4 weeks to PI-8 weeks. The persistence of expression after non-viral gene delivery has historically been challenging; however, in other studies, we have utilized vector engineering strategies to develop gene expression cassettes optimized for long-term, persistent gene expression in the retina and RPE. For example, our vectors carrying scaffold/matrix attachment region (S/MAR) regions promote long-term episomal maintenance of DNA and have led to prolonged gene expression in the mouse retina for up to 15 months post-injection.^23^ Future use of the HA-NS for retinal gene delivery will likely take advantage of these highly optimized vectors.

An interesting fact about the HA-NS is the ability to carry different payloads such as small molecular drugs or proteins besides DNA. The payload concentration that can be loaded and the release kinetics need to be optimized for each formulation. This speaks for the versatility of HA-NSs as vehicles for retinal delivery. Future plans will focus on testing multiple doses, repeated applications, and long-term toxicity evaluations.

Fundus imaging in live animals to detect GFP-expressing cells is much less sensitive compared to the immunohistochemistry technique, likely requiring higher expression levels for detection. The challenge in visualizing the signal through fundus imaging is compounded by GFP expression in the RPE, further complicating observation. This clarification has been included in the discussion.

In conclusion, we here report robust and widespread outer retina gene expression after intravitreal injection of HA-NSs. The ability to reach the outer retina after intravitreal injection is an exciting advancement that expands the potential options available for targeting the multitude of IRD genes in the photoreceptors and RPE.

## Materials and methods

### Animal models

Male and female albino (BALB/c) mice were used for this study at post-natal day (P)30–P90. Animals were maintained in 12 light:12 dark cyclic light at ∼30 lux. For terminal experiments, mice were euthanized by CO_2_ inhalation, consistent with the recommendations of the Panel on Euthanasia of the American Veterinary Medical Association. All handling, maintenance, and experimental use of animals followed protocols approved by the Institutional Animal Care and Use Committee at the University of Houston and were performed according to the NIH and the Association for Research in Vision and Ophthalmology guidelines.

### Fabrication of CBA-GFP-loaded nanoparticles

To a sodium hyaluronate (Aldrich, molecular weight [MW] 30,000–50,000, 2%) solution in deionized water (dH_2_O), CBA-GFP (800 μg in 200 μL PBS) DNA was added and vortexed to form a homogeneous solution. To this solution, chitosan (Aldrich, MW 190,000–375,000, 1% in dH_2_O, 1 mL) was dropwise added with vigorous vortexing for 5 min. A cloudy solution was formed, which was centrifuged at 14,000 rpm for 10 min at 4°C to form a pellet and washed with dH_2_O or PBS. The pellet was resuspended in dH_2_O or PBS and vortexed for a minute, followed by probe sonication for 30 s to 1 min to obtain a homogeneous suspension, which was filtered through a 1 μm filter to remove large aggregates. The nanoparticle suspension thus prepared was used for the described studies.^25^

### Gel electrophoresis of HA-NS

After dispersing HA-NSs by vortexing, 1–3 μL NSs were diluted to 10 μL with sterile water and 2 μL of 6× loading buffer was added. Samples were then loaded on a 0.8% agarose gel alongside a 1 kb Plus DNA ladder (10787018, Invitrogen, Carlsbad, CA, USA). After electrophoresis, the gel was imaged using a ChemiDoc TM MP imager (Bio-Rad Laboratories, Hercules, CA, USA). HA-NS-empty were used as a control.

### Assessments by DLS

After dispersing the NSs by vortexing, 10 μL HA-NSs were diluted in 1,490 μL sterile water (GL1629, Hospira, Lake Forest, IL, USA), and 50 μL of the diluted sample was analyzed by DLS for particle sizing and distribution of the NSs. The remaining amount of the diluted HA-NS was used for zeta potential analysis. DLS and zeta potential analyses were performed using the Nanobrook Omni (Brookhaven Instruments, Holtsville, NY, USA). These tests were performed three times per NS.

### NS fluorescent image

NSs were loaded with FITC-dextran for imaging purposes, and 25 μL of the fluorescent sample was used for imaging using a Zeiss Axiophot microscope.

### Cell transfection

HEK293 cells were seeded in a 96-well plate and transfected at a confluence of 60%. Transfection of both naked DNA and NSs (HA-NS-high) was conducted using lipofectamine 3000 (L3000008, Thermo Fisher Scientific, Waltham, MA, USA) according to the manufacturer’s instructions. 1 μg of naked DNA or the equivalent HA-NS was used for the transfection. Following transfection, cells were supplied with fresh DMEM medium (12800-017, Thermo Fisher Scientific) and incubated for 24 h. After incubation, cells were imaged using a fluorescence microscope (Leica DMIL LED Fluo, Leica Microsystems, Wetzlar, Germany) to visualize GFP expression. HA-NS-empty were used as negative control.

### Preparation of ST solution

ST was purchased from BACHEM (Torrance, CA, USA, H-Tyr(SO3H)-OH sodium salt Cat#4033889, Lot#1056704) and prepared as described previously.^21^ Briefly, the ST was dissolved in endotoxin-free 0.9% saline at the desired concentrations at 70°C. Working solutions of ST were mixed with equal volumes of NSs by tapping gently immediately before injections. As controls, equal volumes of NSs were mixed with saline prior to injection.

### Intravitreal injections

Intravitreal injections were performed as described previously.^21^ Within a sterile surgical room, adult BALB/c mice were anesthetized by an intraperitoneal injection of 85 mg/kg ketamine and 14 mg/kg xylazine (Henry Schein Animal Health, Dublin, OH, USA). Eyes were dilated with 1% cyclopentolate (Bausch and Lomb, Rochester, NY, USA). A very shallow puncture was made through the sclera in the dorsal hemisphere using a beveled 30G needle (BD Biosciences, Franklin Lakes, NJ, USA). Through this puncture, a 33G blunt-end needle was introduced into the vitreous cavity, and 1.5 μL of material (NSs, DNA, or ST/HA-NS mixture) was injected into the vitreous space near the optic nerve using a 5 μL microinjection syringe (Hamilton, Reno, NV, USA).

### Fundus imaging

Fundus imaging was performed using the Micron IV system (Phoenix Research Laboratories, Pleasanton, CA, USA) as described previously.^14^^,^^21^ Animals were anesthetized/dilated as for injections. Bright-field images were captured first, and then GFP expression was analyzed by imaging with the green filters (451.5–486.5 nm excitation and 488 nm emission, for fluorescein). All images were captured using StreamPix software (Phoenix Research Laboratories, Pleasanton, CA, USA).

### ERG

Full-field ERGs were recorded as described previously.^26^ Animals were dark adapted overnight. Prior to ERG, they were anesthetized using ketamine and xylazine (85 and 14 mg/kg, respectively, Henry Schein Animal Health). Eyes were dilated with 1% cyclopentolate and covered in Gonak. ERGs were recorded using platinum loop electrodes on the cornea. Using the UTAS system (LKC, Gaithersburg, MD, USA), dark-adapted full-field scotopic ERG responses were recorded from each eye in response to a single 157 cd s/m^2^ flash. Animals were light adapted for 5 min (29.03 cd/m^2^), and then photopic ERG responses were recorded in response to a series of 25 flashes at 79 cd s/m^2^.

### Fluorescence imaging and signal intensity calculation

After euthanasia, the whole eye was enucleated, cryoprotected, and embedded without fixation. Eyes were cryosectioned along the superior-inferior plane from temporal to nasal, and 10-μm-thick sections were collected every ∼200 μm. Sections were imaged immediately after collecting without further processing or mounting. Stitched images covering the whole retinal section were generated by 20× (0.8 air) tile imaging using the Zeiss Confocal Microscope (Zeiss, Jena, Germany) and Zen 3.2 Blue software. GFP gene expression was imaged on the green channel (excitation peak at 488 nm and an emission peak at 509 nm). HA-NS localization was visualized on the red channel (excitation peak at 553 nm and an emission peak at 627 nm).

To generate graphical maps estimating HA-NS and GFP distribution in the retina, six regions (600,000 μm^2^) from the superior to the inferior plane (corresponding to regions 1–6) were created from each of the five separate retinal sections along the nasal to temporal axes as in Eblimit et al.^21^ (refer to Figure S5). Calculation of the signal intensity for TxRed and GFP was done using the ZEN 3.2 Blue software. A selection tool was used to highlight a region of interest containing the retina from the ILM to the RPE, avoiding the sclera due to the greater autofluorescence in that tissue. The total signal intensity of either TxRed or GFP in each image was calculated by multiplying each pixel signal intensity by its frequency and then summing all the individual values. To keep all images comparable, the laser strength and all imaging/analysis software settings were kept the same for all samples. In graphical maps/heatmaps, white squares represent images containing less signal intensity, while either red (TxRed) or green (GFP) squares contained more signal intensity. Heatmap intensities were calculated with all PI-4 week eyes together so that color intensity could be compared across eyes for a given time point. Similarly, intensities were calculated with all PI-8 week eyes together.

For nuclei counts, sections were post-fixed in 4% paraformaldehyde for 5 min, washed for 10 min in 1× PBS, stained with DAPI for 30 min, washed with 1× PBS for 10 min 3 times, and mounted in ProLong gold anti-fade mounting media (Thermo Fisher Scientific) prior to imaging as described above. ImageJ software was used to count the number of nuclei.

### Flat mounts

Eyes were enucleated, immediately fixed in 4% paraformaldehyde for 5 min at room temperature, and a puncture was made in the cornea to allow better penetration of the fixative over another 2 h. Eyes were then dissected to remove the cornea and lens and left to fix further for another 30 min and then washed in 1× PBS. The RPE was dissected away from the neural retina, and both pieces were separately flat mounted by making four relaxing incisions and laying them flat. Flat mounts were incubated with anti-GFP antibody (488 conjugated, A-21311, Invitrogen) and DAPI overnight at 4°C. The flat mounts were then washed four times for 15 min in 1× PBS. Then, using Fluoroshield with DAPI (Sigma-Aldrich, St. Louis, MO), flat mounts were mounted on a glass microscope slide. For initial imaging, 5× tile images were taken of the whole tissue. Higher-magnification images were taken using 63× magnification on a Leica Stereo microscope (Model MDG41, Leica Microsystems, Deerfield, IL, USA) from various areas in the tissue.

Quantification of fluorescence in flat mounts was performed by importing captured Zen files into ImageJ for analysis. Each image was converted to an 8-bit image, and the color was inverted. Images from eyes injected with vehicle only were used to establish a thresholding that would eliminate the background signal and capture only the signal from the injected particulate. This threshold was applied to every image, and mean intensity values were generated using ImageJ’s measure tool.

### Statistical analysis

Statistical analysis was performed using Graphpad Prism v.9.2. Differences between groups were analyzed using two-way ANOVA with Tukey’s post hoc comparison.

## Data and code availability

All data associated with this study are presented in the paper or the supplemental information.

## Supplemental information

Document S1. Figures S1–S11

Document S2. Article plus supplemental information
