# Supplementary material for: Effective intravitreal gene delivery to retinal pigment epithelium with hyaluronic acid nanospheres
Source: Mol Ther Nucleic Acids. 2024 May 20;35(2):102222. doi: 10.1016/j.omtn.2024.102222 (PMC11168490; doi:10.1016/j.omtn.2024.102222)
Supplement: Document S1. Figures S1–S11 [file mmc1.pdf]

OMTN, Volume *35*

Supplemental information

Effective intravitreal gene delivery

to retinal pigment epithelium

with hyaluronic acid nanospheres

Ryan Crane, Mustafa S. Makia, Stephanie Zeibak, Lars Tebbe, Larissa Ikele, Christian Rutan Woods, Shannon M. Conley, Ghanashyam Acharya, Muna I. Naash, and Muayyad R. Al-Ubaidi

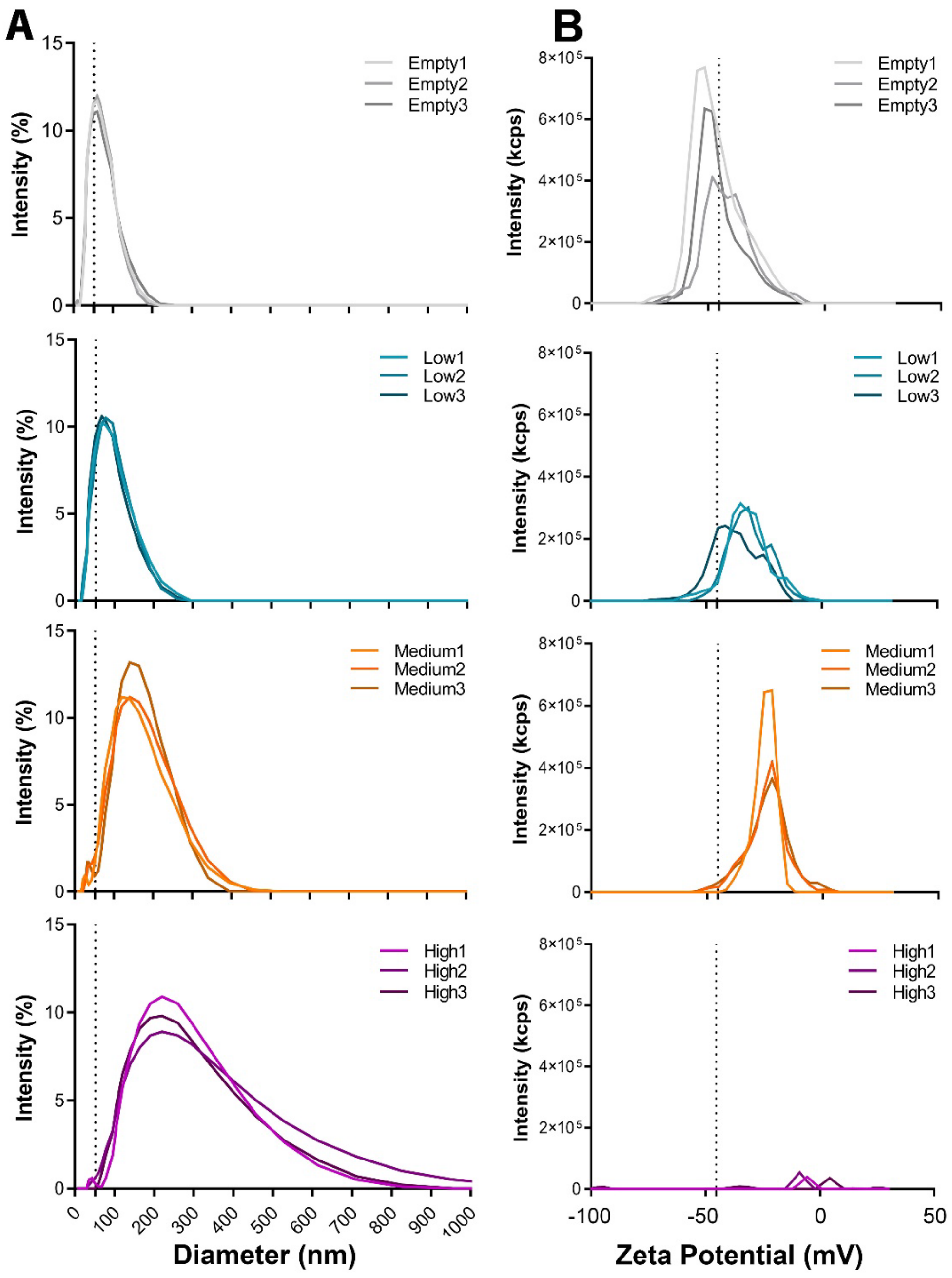

Figure S1. Dynamic light scattering assessments of HA-NS either empty or loaded with different amounts of DNA. A. Changes in HA-NS diameter as a function of increased amounts of DNA. **B.** Shift in Zeta potential as a function of increased amounts of DNA. Dotted lines are placed as a reference to the shift in values relative to empty HA-NS.

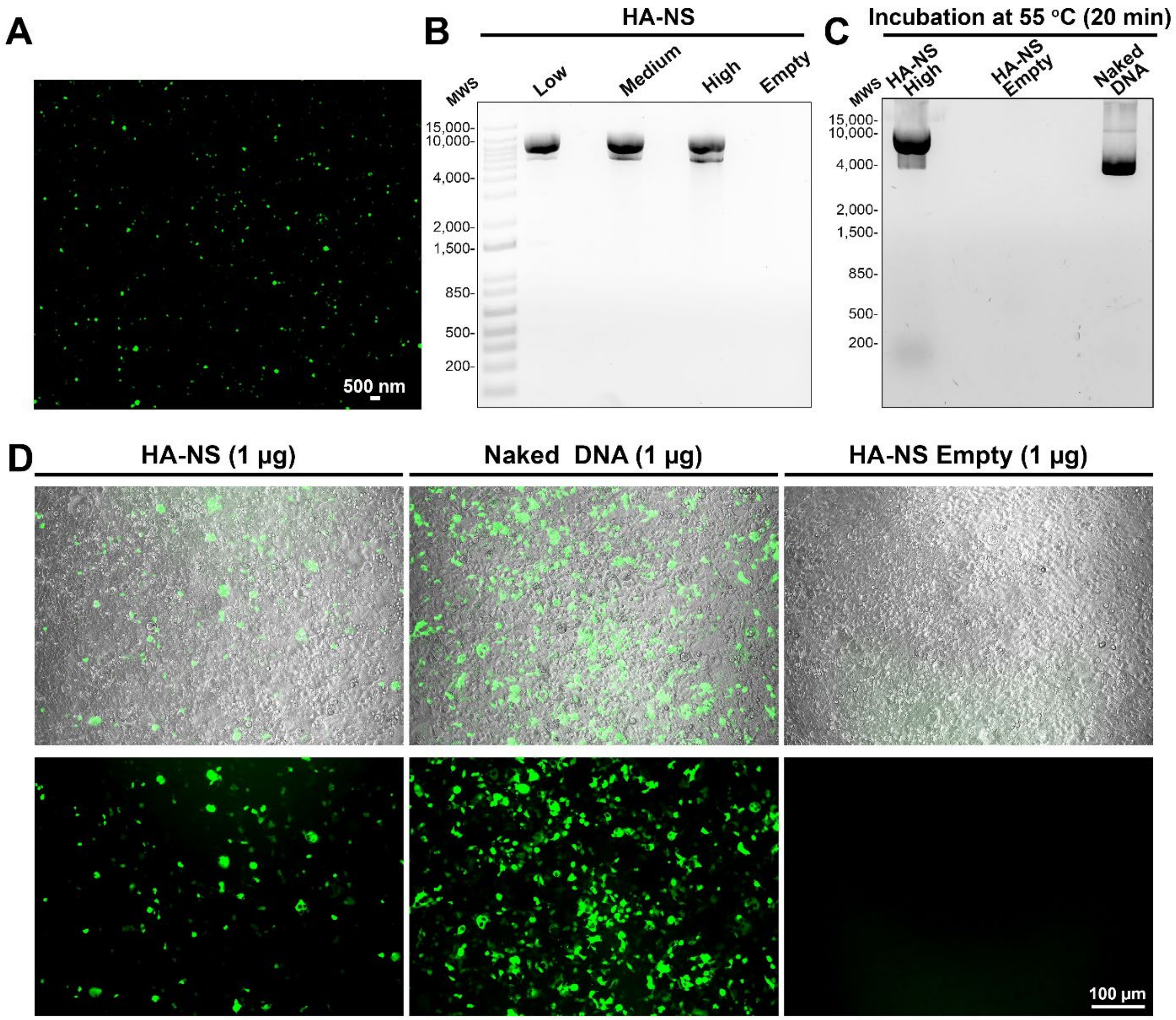

Figure S2. Characterization of HA-NS. A. Twenty-five microliters of HA-NS filled with FITC-dextran were spotted on a microscope slide, cover-slipped and imaged using a Zeiss Axiophot microscope. **B.** Empty HA-NS or filled with different amounts of DNA were separated on 0.8% agarose gel electrophoresis. **C.** HA-NS-high and empty nanospheres (HA-NS empty) were subjected to a 20 min incubation in 1xPBS at 55ºC. Naked DNA was used as control. **D.** HEK293 cells transfected with HA-NS-high and naked DNA using lipofectamine 3000. Upper panel: GFP fluorescence superimposed on bright-field image of the cells. Empty HA-NS were used as negative control. Lower panel: GFP expression following transfection.

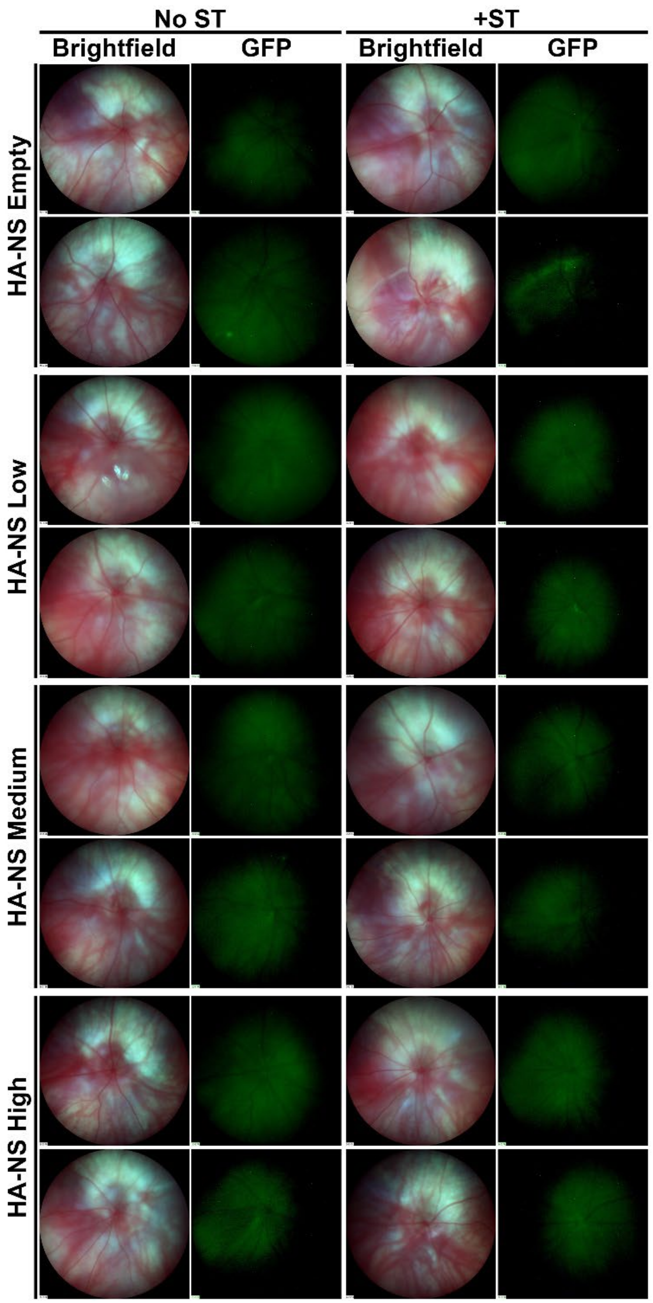

Figure S3. Fundus images at PI-4 weeks following IV NS injection. Mice injected with HA-NS either with or without ST underwent fundus imaging at PI-4 weeks. In each pair of images, the brightfield fundus is on the left and the GFP channel is on the right. Minimal GFP expression was observed. Both eyes from three animals were imaged and used for the analysis per group.

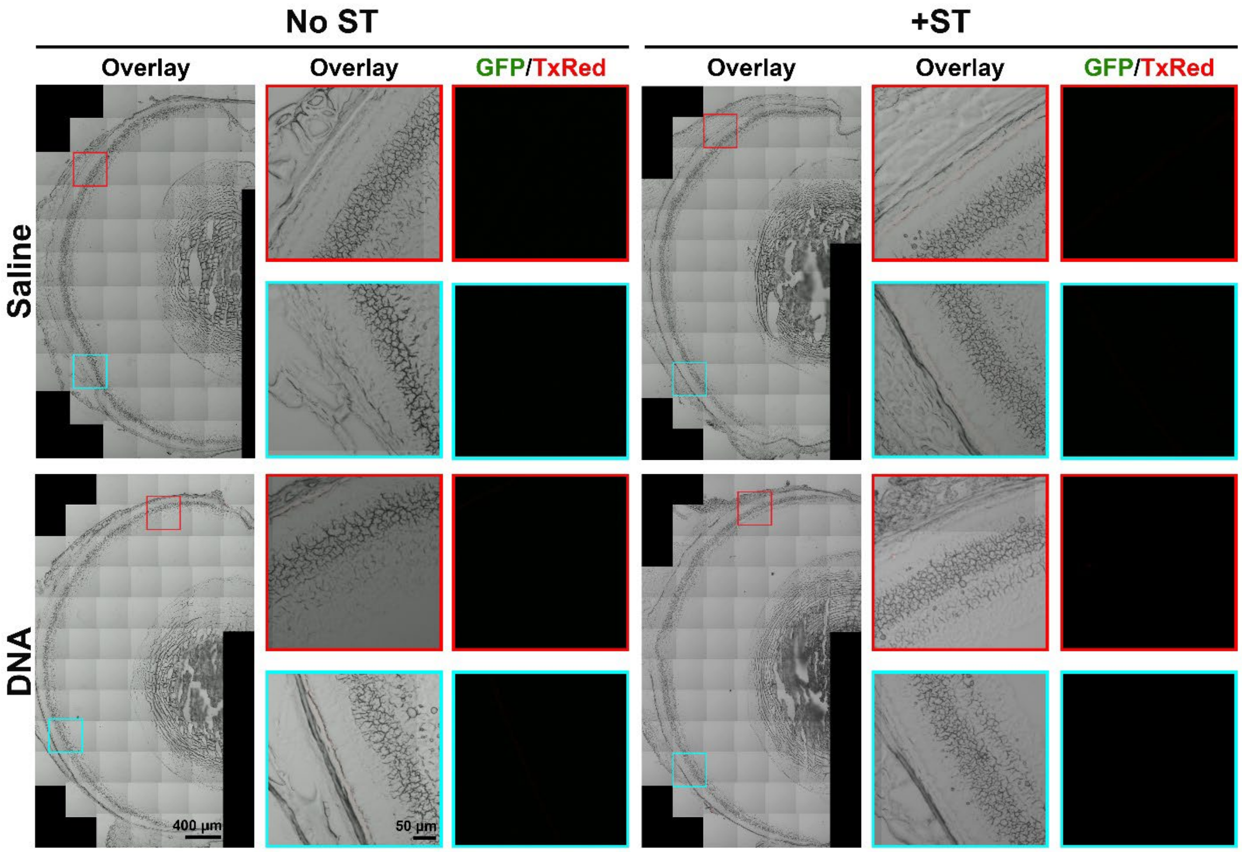

Figure S4. Naked DNA and saline do not lead to TxRed or GFP signal. Eyes were collected from mice injected with naked DNA (no HA-NS) or saline (vehicle) at PI-4 weeks. Eyes were harvested, cryosectioned, and imaged for native fluorescence. The left panel of each image set shows brightfield, GFP, and TxRed signal, red and blue boxed areas are shown larger in the middle and right panels. No visible fluorescence is observed in the TxRed or GFP channels. Both eyes from three animals were imaged and used for the analysis per group. Scale bars 400 µm on overview images and 50 µm for insets.

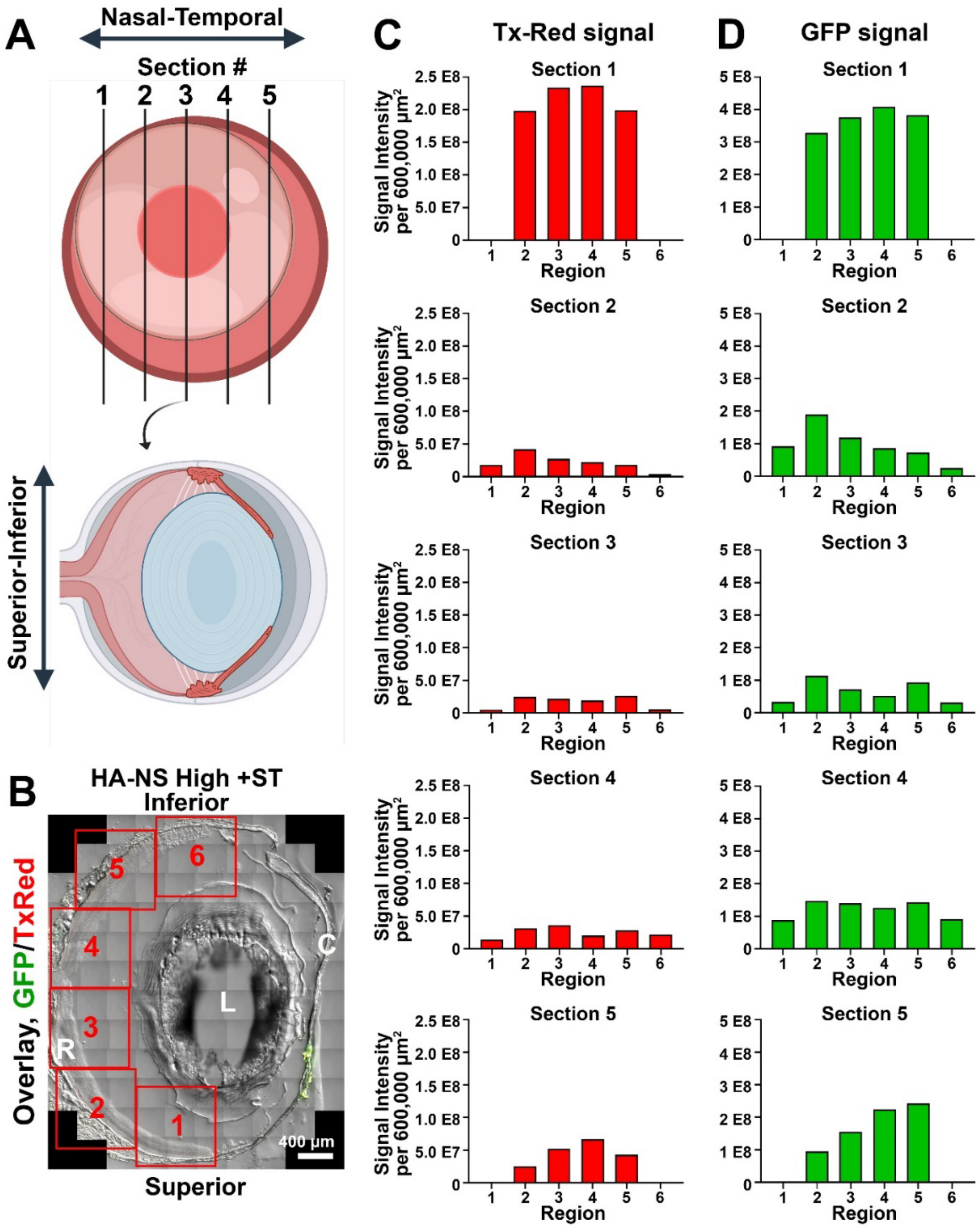

Figure S5. Schematic for calculating signal intensity from HA-NS-injected native cross section. A. Schematic illustrating where sections were captured across the eye from nasal to temporal. **B.** Representative cross section, broken into 6 imaging regions from superior [1] to inferior [6]. **C-D.** Example graphs plotting the amount of TxRed (**C**) or GFP (**D**) fluorescence in each region in each section. These fluorescence values were used to generate heat maps presented throughout the manuscript. R: retina, L: lens, C: cornea.

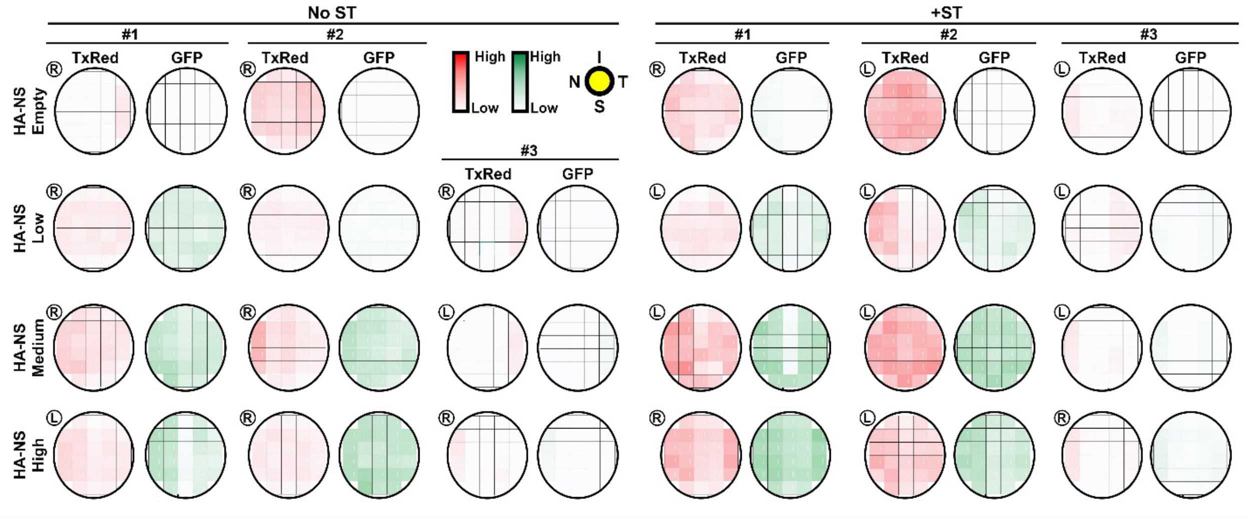

Figure S6. Co-delivery of ST with HA-NS leads to gene expression at PI-4 weeks. Adult mouse eyes were injected with 1.5 µl of HA-NS Empty, HA-NS Low, HA-NS Medium, or HA-NS High either without, or with ST. Tissues were collected at PI-4 weeks and sectioned as in **Fig. S5**. HA-NS TxRed fluorescence intensity and GFP fluorescence intensity were measured in adjacent regions throughout a retinal section and in multiple sections throughout the eye. Each row corresponds to a section, and each box corresponds to a region in that section refer to **Fig. S5**. Shown are maps 2-3 additional eyes per group, colored using the same criteria as those shown in **Fig 2**. Intensity of color corresponds with increasing fluorescence intensity. R: right eye, L: left eye, I: inferior, S: superior, N: nasal, T: temporal.

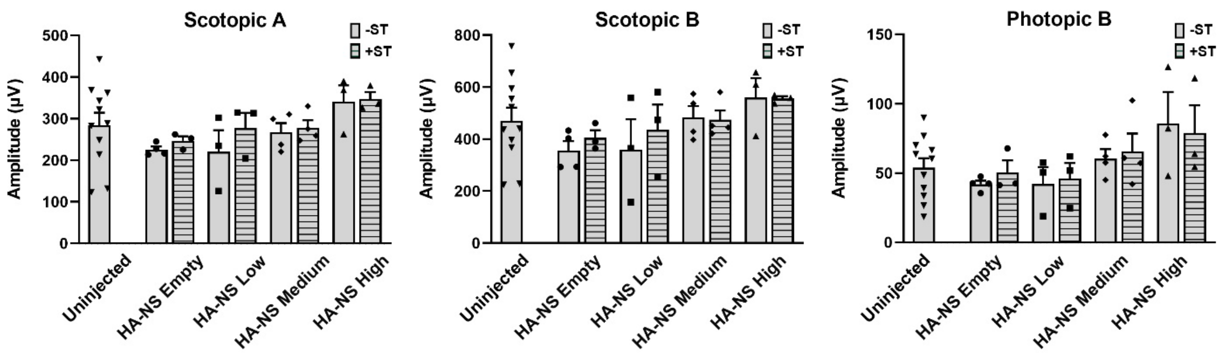

Figure S7: HA-NS do not elicit significant changes in ERG function in the mouse eye. Eyes were injected with HA-NS as labeled and underwent full-field ERG recordings under scotopic (left and middle) and photopic (right) conditions at PI-4 weeks. Plotted is mean ERG amplitude ± SEM. Each spot represents one eye. Both eyes from three animals were tested and used for the analysis per group, both eyes were then averaged together for an n of one per animal. There were no significant differences between groups by one-way ANOVA.

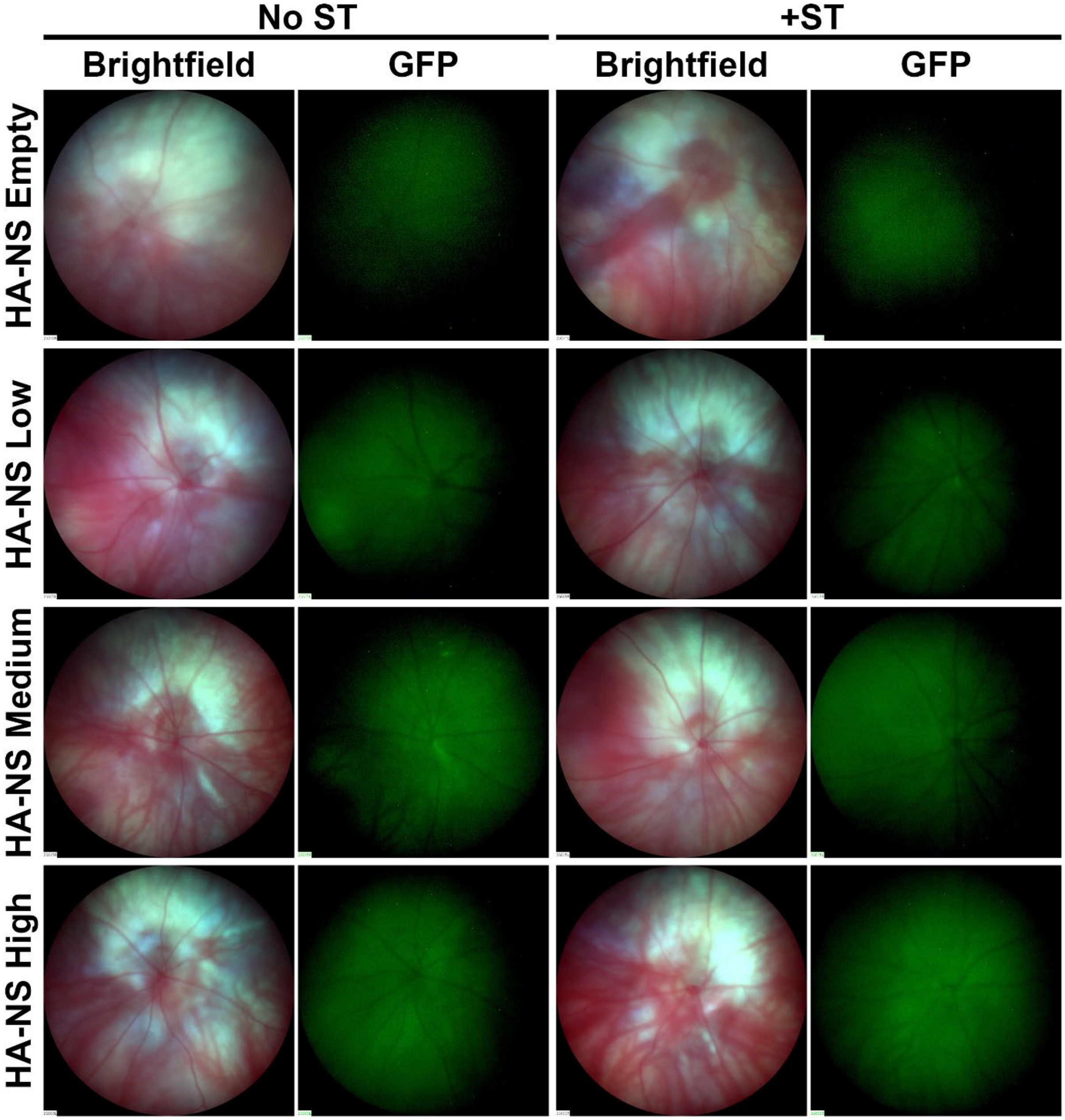

Figure S8. Fundus images at PI-8 weeks following IV NS injection. Mice injected with HA-NS either with or without ST underwent fundus imaging at PI-8 weeks. In each pair of images, the brightfield fundus is on the left and the GFP channel is on the right. Minimal GFP expression was observed. Both eyes from one animal were imaged and used for the analysis per group.

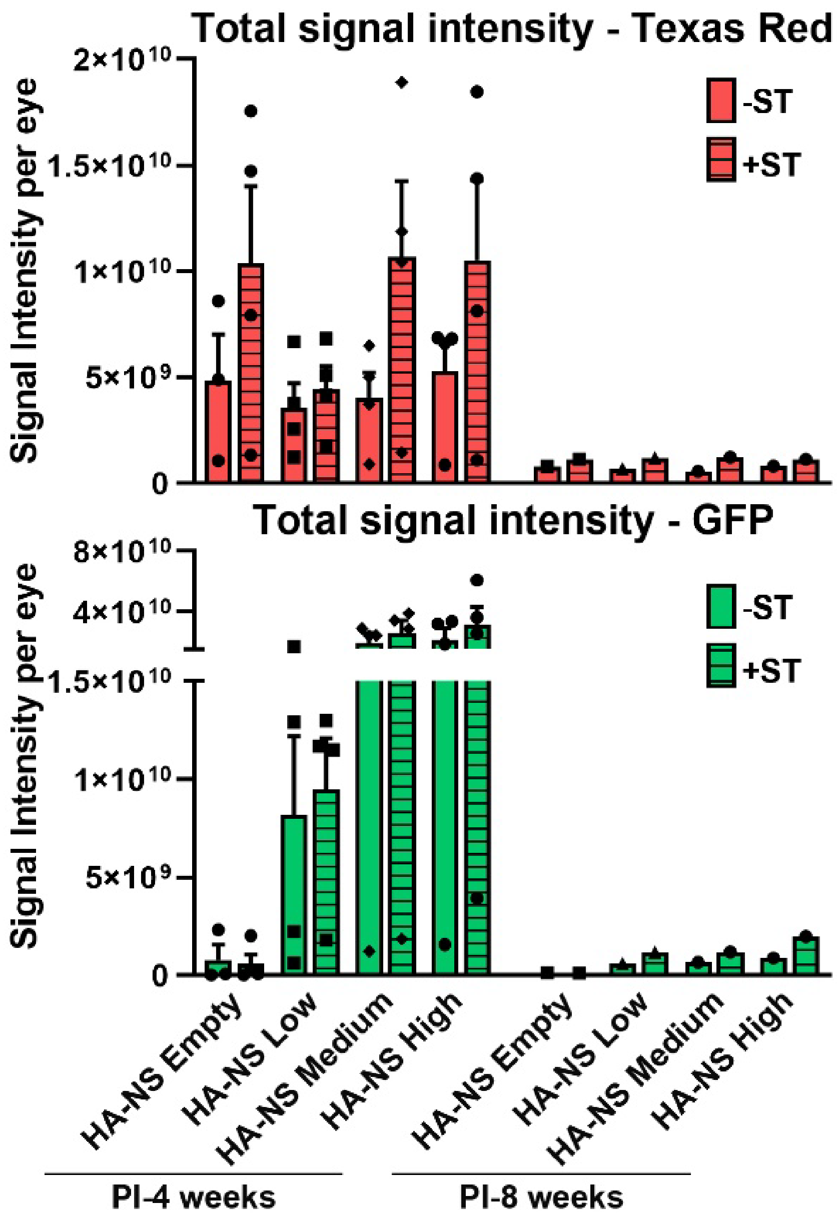

Figure S9. HA-NS TxRed and GFP intensity drop from PI-4 to PI-8 weeks. Shown are total eye fluorescence data (top is TxRed, and bottom is GFP) replotted from Fig. 2 and 5 with both timepoints together and the same scale on the Y-axis. Each spot represents one eye.

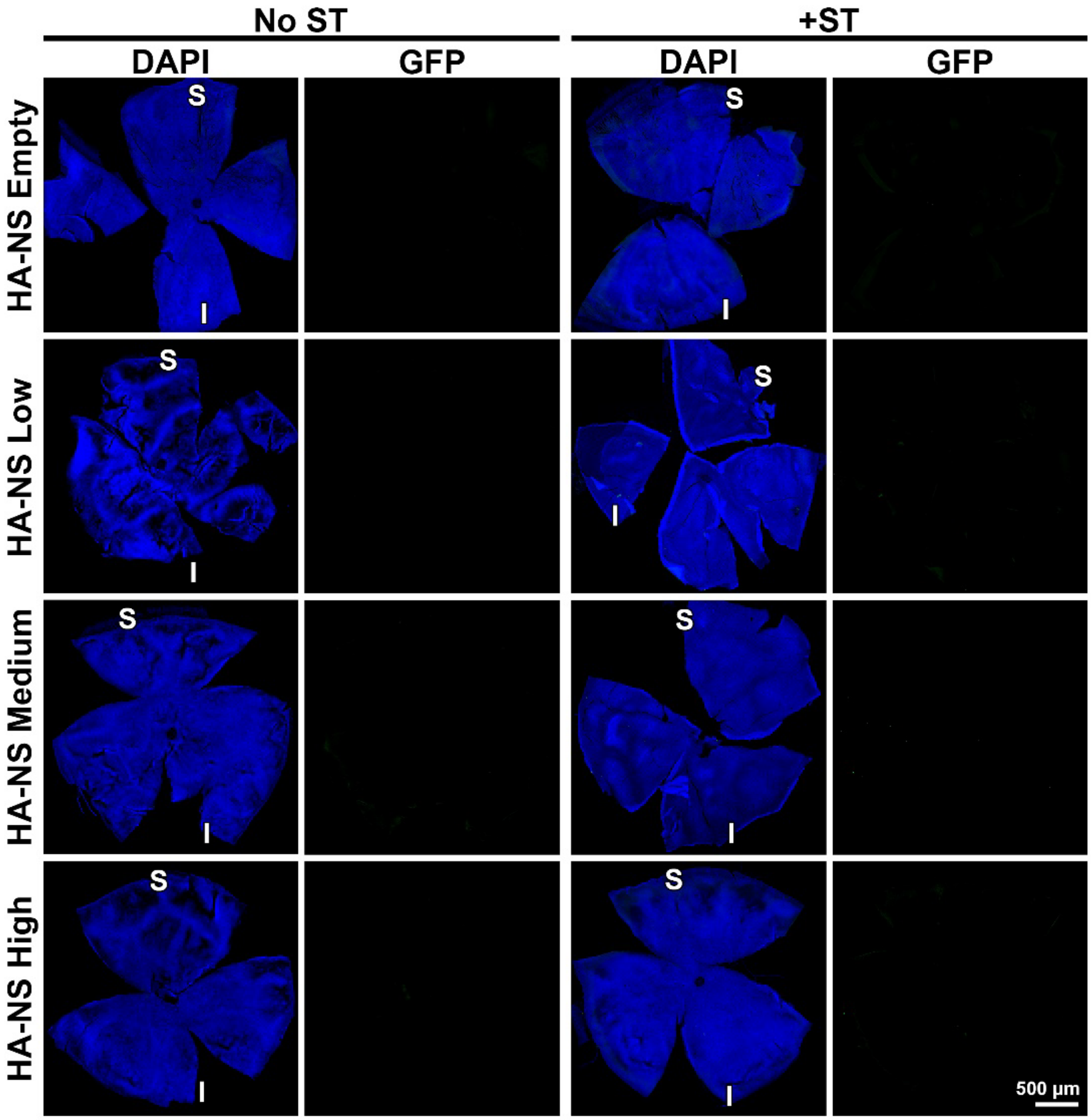

Figure S10. The retina does not express detectable GFP after injection with HA-NS. Adult mouse eyes were injected with HA-NS Empty, HA-NS Low, HA-NS Medium, or HA-NS High either without or with ST and tissues were harvested at PI-8 weeks. Shown are retinal flat mounts that were labeled with GFP antibodies (right panels) and counterstained with DAPI (blue, left). One eye from one animal was collected and imaged per group. Scale bar: 500 µm. S: superior, I: inferior.

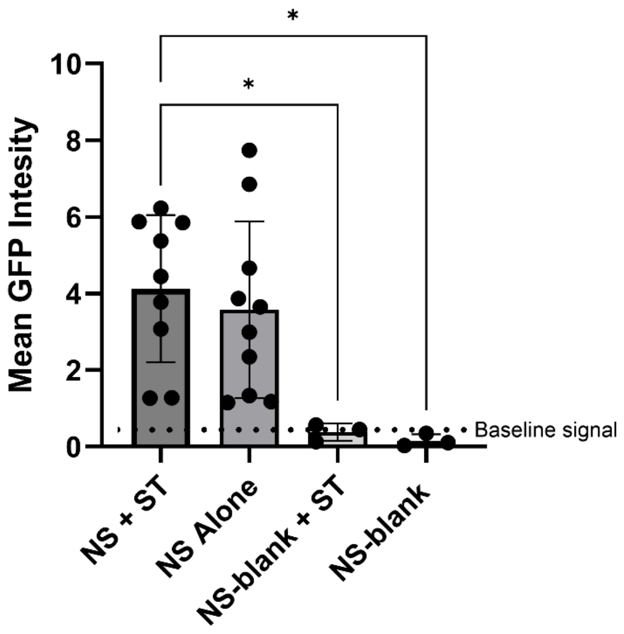

Figure S11. Mean Fluorescent Intensity of RPE flatmounts of NS-injected Eyes. Mean fluorescent intensity was assessed from similar regions of interest isolated from RPE flatmounts. Intensities were measured from eyes injected with GFP-labeled NS in the presence and absence of sulfotyrosine, along with corresponding controls. Intensity values are presented as the mean ± SD. N=3 for each condition, and N=1 for experimental controls. One-way ANOVA followed by Tukey-Kramer's post-hoc testing was used to determine statistical significance. Three images were captured from each sample using a 63x objective, with 1.3x zoom. * is $P \leq 0.05$
